# IGF-1’s protective effect on OSAS rats’ learning and memory

**DOI:** 10.1007/s11325-024-03047-8

**Published:** 2024-06-11

**Authors:** Ling Zeng, Ting Yu, Haijun Liu, Mi Li, Jin Wang, Changsheng Wang, Ping Xu

**Affiliations:** 1 Department of Neurology, Affiliated Hospital of Zunyi Medical University, 149 Dalian Road, Zunyi, Guizhou 563000 China; 2 Key Laboratory of Basic Pharmacology of Ministry of Education, Zunyi Medical University, Zunyi, Guizhou 563000 China; 3 Critical Care Medicine, Affiliated Hospital of Zunyi Medical University, 149 Dalian Road, Zunyi, Guizhou 563000 China

**Keywords:** OSAS, Cognitive, IGF-1, SYP, Memory

## Abstract

**Purpose:**

Patients with obstructive sleep apnea syndrome (OSAS) frequently experience cognitive dysfunction, which may be connected to chronic intermittent hypoxia (CIH). Insulin-like growth factor-1 (IGF-1) is thought to be closely associated with cognitive function, but its role in cognitive impairment caused by OSAS is unclear. The purpose of this study was to investigate the potential protective effect of IGF-1 on cognitive impairment in OSAS rats.

**Methods:**

Healthy male SD rats (*n* = 40) were randomly assigned into four groups: control group, CIH group, NS + CIH group, and IGF-1 + CIH group. All experimental rats except for those in the control group were exposed to intermittent hypoxic (IH) environments for 8 h per day over 28 days. Prior to daily exposure to IH, rats in the IGF-1 + CIH group received subcutaneous injections of IGF-1. The Morris water maze test was conducted on all experimental rats. Brain tissue testing methods included Enzyme-Linked Immunosorbent Assay, Hematoxylin and eosin staining, Immunohistochemistry, and Western blotting.

**Results:**

The rat model of OSAS was successfully established following exposure to CIH and exhibited significant cognitive impairment. However, daily subcutaneous injections of IGF-1 partially restored the impaired cognitive function in OSAS rats. Compared with the control group, there was a significant decrease in the expression levels of IGF-1, p-IGF-IR, and SYP in the CIH group; however, these expression levels increased significantly in the IGF-I + CIH group.

**Conclusion:**

In OSAS rats, IGF-1 enhances learning memory; this effect may be linked to increased p-IGF-1R and SYP protein production in the hippocampus.

## Introduction

Obstructive sleep apnea syndrome (OSAS) is a common chronic sleep respiratory condition characterized by periodic hypoxemia and fragmentation of sleep due to recurrent apnea and hypopnea during sleep [1]. Long-term OSAS can lead to functional impairment of multiple system organs throughout the body, among which cognitive impairment is one of the common complications of OSAS, which affects the career, family, and social life of patients to a certain extent, reducing the quality of life, and is also one of the important reasons for sudden death and traffic accidents in OSAS patients, becoming one of the public health problems paid attention to around the world [2, 3]. Recurrent hypoxemia during sleep is thought to be the primary cause of cognitive impairment, even if the precise mechanism behind the cognitive dysfunction brought on by OSAS remains unclear.

The synapse is an essential component of the brain that facilitates information transmission and is essential to memory and learning. SYP is a specific marker protein of presynaptic synaptic vesicles, and its density and distribution can indirectly reflect the number and distribution of synapses in the body, which is closely related to cognitive processes [4]. Studies have found that CIH can reduce the length of the synaptic active region and the number of dendritic spines in the hippocampus and affect long-term enhancement and synaptic remodeling, which is believed to be closely related to cognitive dysfunction caused by OSAS [5]. Nevertheless, it is still unknown how SYP is expressed when OSAS-induced cognitive impairment first appears and worsens.

Growth factors such as IGF-1 are essential for many biological functions, such as cell division, growth, and metabolism. During the development of the brain, it promotes the growth and differentiation of neurons by activating specific receptors and downstream signaling pathways [6, 7]. In addition, IGF-1 has been found to affect synaptic plasticity, enhance synaptic density, and promote communication between neurons, so it is believed to be closely linked to the maintenance of cognitive functions [8, 9]. The risk and development of cognitive diseases, including dementia and Alzheimer’s disease, have been associated with levels of IGF-1. IGF-1 has been found at lower levels in patients with various illnesses, and the severity of the disease is adversely connected with IGF-1’s levels. Furthermore, studies suggest that increasing IGF-1 levels in the body could slow down or improve cognitive decline associated with Alzheimer’s disease [10]. It is currently unknown, nevertheless, if IGF-1 can treat OSAS-related cognitive impairment.

The present body of data supports the significance of IGF-1 through its effects on synapses in sustaining cognitive health, even though the precise mechanisms and linkages between IGF-1 and synaptic plasticity remain a matter of discussion.

In this study, we found that consistent with previous studies, the rats exposed to CIH showed significant cognitive impairment, suggesting that CIH is suitable for OSAS models with cognitive impairment. In addition, we also found that subcutaneous injection of IGF-1 can improve the cognitive dysfunction of OSAS rats and increase the expression of SYP in the hippocampus of rats, suggesting that the protective effect of IGF-1 on cognitive function may be achieved through the regulation of SYP and synaptic plasticity.

## Materials and methods

### Establishment of the intermittent hypoxia rat model

Healthy SPF-grade male SD rats (160-180 g, *n* = 40) were obtained from the Animal Experiment Center of the Chinese Army Medical University (license number: SCXK (Chongqing) 2015–0005). All rats were housed in rooms with a constant temperature of 22–24 °C, a 12-h light–dark cycle, and ad libitum access to food and water. All procedures were approved by the Animal Care and Use Committee of Zunyi Medical University, Zunyi, China(approval number [2016]2–045).

After 1 week of adaptive feeding in the same environment, 40 rats were randomly divided into four groups, namely the control group, CIH group, NS + CIH group, and IGF-1 + CIH group, with 10 rats in each group. The CIH, NS + CIH group and IGF-1 + CIH group were exposed to intermittent hypoxic (IH) environments. 90 s are anoxic (oxygen concentration was 8%-10%), and the remaining 90 s are normal-oxygen(oxygen concentration was 21%), alternated for 8 h per d for 28 d. Before receiving intermittent hypoxia daily, rats in the IGF-1 + CIH group received a subcutaneous injection of IGF-1 (50 μg/kg, dissolved in 0.9% saline, PeproTech, USA), while rats in the NS + CIH group received a subcutaneous injection of the same volume of 0.9% normal saline.

### Morris water maze

The Morris water maze (MWM) is a circular pool with a diameter of 200 cm, with black walls enclosed by a black shade fabric to block out light and maintain silence during the experiment. The pool is divided into four quadrants by a line drawn across its center along both the horizontal and vertical axes. A circular movable platform (escape platform) with a diameter of approximately 15 cm is placed in the target quadrant of one of the pool’s quadrants for the positioning navigation experiment. For the spatial exploration experiment, the hidden platform is removed, while other conditions remain unchanged.

MWM, conducted over 7 d, consists of two parts: positioning navigation and spatial exploration. To acclimate to the environment and minimize the effects of stress on the experiment, on the first d, the rats swim in the pool for two min with their heads facing the pool wall.

For the positioning navigation experiment on d 2–6, the rats are placed in the water with their heads facing the pool wall. Their movements are observed using video tracking software, and the time taken by the rats to find the hidden platform is recorded. This duration is termed the escape latency period. If the platform is not found within 120 s, the rats are guided to the platform and allowed to remain there for 10 s to help them remember its location.

For the spatial exploration experiment on d 7, the hidden platform is removed from the pool. The rats are then immersed in the water with their heads facing the pool wall. Their movements are observed, and the number of times they actually cross the original platform area within 120 s is counted.

### Tissue preparation

After MWM, rat brain tissue samples were collected, and intraperitoneal anesthesia was performed using 10% chloral hydrate per kilogram of body weight. Following anesthesia, the thoracic cavity and pericardium of rats (*n* = 4 per group) were incised to expose the heart and ascending aorta. Blood was observed flowing out after incising the right atrium. Subsequently, 300 ml of saline was rapidly injected into the perfusion needle, followed by perfusion fixation using 300 ml of 4% paraformaldehyde. After removing the brain tissue, it was preserved in 4% paraformaldehyde fixative for 24–48 h before being sent to the pathology department of the affiliated hospital of Zunyi Medical College for paraffin embedding and sectioning (4 mm). The hippocampal tissue of the rest of the rats (*n* = 6 per group) was quickly separated on ice, wrapped in tin foil, placed in EP tubes, and promptly transferred to -80℃ for freezing and storage.

### Enzyme-linked immunosorbent assay

After grinding the rat hippocampus tissue, the supernatant was collected after centrifugation at 15,000 rpm for 30 min at 4 °C. Enzyme-linked immunosorbent Assay (ELISA) was used to measure the expression level of IGF-1 protein in the rat hippocampus. The ELISA kit was purchased from Immunoway (KE1432) in the United States, and the procedure was conducted according to the instructions provided with the kit.

### Hematoxylin and eosin staining

The paraffin-embedded brain tissue sections (*n* = 4 per group) were subjected to deparaffinization and dehydration processes. Subsequently, antigen retrieval was performed in citrate buffer, followed by staining with hematoxylin and eosin. After completion, the sections were sealed with neutral gum and observed under a microscope.

### Immunohistochemistry

The paraffin-embedded brain tissue sections were deparaffinized, followed by antigen retrieval using citrate buffer. The brain sections were then incubated with primary antibodies for 12 h: p-IGF-1R (1:50, Abcam, USA) and SYP (1:400, Abcam, USA). Finally, they were incubated with sary antibodies for 30 min, stained with diaminobenzidine, restained with hematoxylin, differentiated with 0.1% HCl, dehydrated with gradient alcohol, and finally fixed and observed.

### Western blotting

According to the instructions of the whole protein Kit (Kaiji Biological Technology, Jiangsu, China), the rat hippocampal tissue stored at -80 °C was removed for protein extraction and quantification. Denatured protein samples (10 μl) and Mark (5 μl) were added to the prepared sodium dodecyl sulfate–polyacrylamide gel (SDS-PAGE, Leagene, Beijing, China) for electrophoresis (80 V). After electrophoretic separation, the target protein was transferred to the prepared PVDF membrane by electric transfer (200 mA; the time of membrane transfer was adjusted according to the molecular weight of the protein). Next, The PVDF membrane was immersed in 5% skim milk at room temperature for 2 h. Then, the PVDF membrane was incubated respectively with different primary antibody dilutions, SYP (1:1000, Abcam, USA), p-IGF-1R (1:500, ImmunoWay, USA), IGF-1R (1:500, ImmunoWay, USA) or β-tubulin (1:5000, Abcam, USA), in a refrigerator shaking bed at 4℃ overnight after being washed with TBST(Bioengineering Corporation, Shanghai, China). On the 2nd d, the PVDF membrane was removed, washed with TBST and incubated at room temperature by the 2nd antibody for 2 h. Finally, PVDF membranes which were covered by the luminous reagent (A:B = 1:1, MILLIPORE, USA), were exposed in automatic exposure instrument (Bio-Rad, USA).

### Statistical methods

The data analysis was conducted using SPSS software (version 17, IBM, Armonk, NY, USA), and the results are expressed as the mean ± standard error of the mean. The escape latency data from the Morris water maze were statistically evaluated using repeated measures analysis of variance, Rat tail artery SpO2 data were analyzed using paired t-tests, and other data were analyzed using Tukey’s one-way analysis of variance.

## Results

### The CIH rat model conformed with the evaluation criteria of the OSAS model

**Fig. 1:**
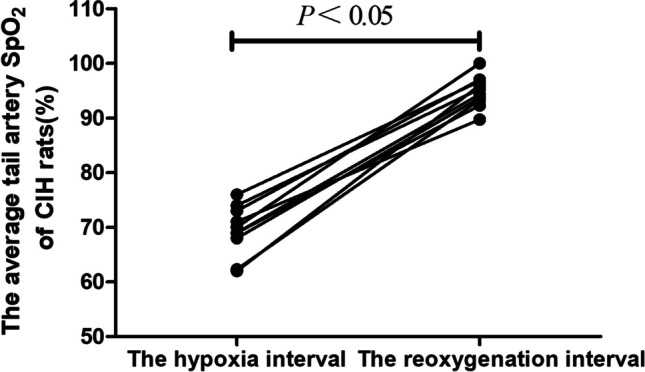
Effect of hypoxia interval and reoxygenation interval on tail artery SpO_2_ of rats (*n* = 10, $\overline{x }$ ± s). The tail artery SpO2 of rats during the hypoxia interval was lower than the reoxygenation interval, and the statistical difference was statistically significant (*P* < 0.05). During the hypoxia and reoxygenation interval, the rat’s tail arterial SpO_2_ was measured three times per rat, and the average of these measurements represented the mean rat tail arterial oxygen saturation during this period

By using the intermittent hypoxia mode in the hypoxic chamber, a rat model that matched the pathophysiological traits of OSAS was created for this work. To obtain 20 hypoxic episodes per h and to guarantee that the IH time reached 8 h each d for 28 d, the rats were forced to IH in 180-s cycles. Rats were observed regularly breathing head-up, snoring, and deepening abdominally during the trial, all of which were consistent with the pathophysiological traits of OSAS. Using a multiparameter monitor to detect the changes in rat tail artery oxygen saturation (SpO_2_). The average tail artery SpO_2_ of rats was 94.8% during the reoxygenation interval and 69.4% during the hypoxia interval. Compared with the reoxygenation interval, the overall range of tail artery SpO_2_ of rats decreased by more than 4%, and the difference was statistically significant. So, OSAS model can be successfully established in rats exposed to CIH (Fig. 1).

### Cognitive deficits could be improved by IGF-1 subcutaneously injection in rats exposed to CIH

**Fig. 2:**
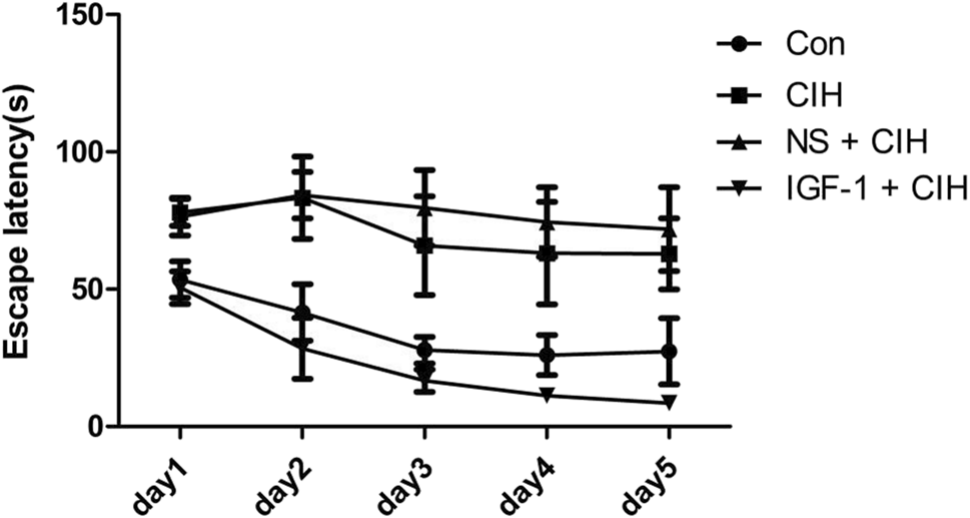
Escape latency of rats in the location-navigation trial (*n* = 10, $\overline{x }$ ± s)

The rats underwent a 5-d location-navigation trial. The escape latency of rats in all groups was shortened to a certain extent as the time went on. Compared with the control group, the escape latency of rats in the CIH group and NS + CIH group was significantly prolonged. However, the escape latency of the IGF-1 + CIH group was gradually shortened with the extension of time, and its trend was even better than that of the control group, although there was no statistical difference (Table 1 and Fig. 2).

**Table 1:** Escape latency of rats in the location-navigation trial (*n* = 10, $\overline{x }$ ± s)

Groups	Time(s)				
	First day	Second day	Third day	Fourth day	Fifth day
Con	53.48 ± 16.34	41.51 ± 25.21	27.82 ± 11.89	26.01 ± 17.76	27.35 ± 29.51
CIH	77.94 ± 12.16^*^	83.24 ± 36.87^*^	65.90 ± 44.09^*^	63.08 ± 45.68*	62.83 ± 31.58^*^
NS + CIH	76.33 ± 16.87^*^	84.20 ± 20.78^*^	79.57 ± 33.72*	74.45 ± 31.l6*	71.86 ± 37.42^*^
IGF-1 + CIH	64.58 ± 19.06^△#^	28.48 ± 27.14^△#^	16.70 ± 9.90^△#^	11.25 ± 3.00^△#^	8.57 ± 3.97^△#^

Compared to the control group, the CIH group and NS + CIH group showed prolonged escape latency (*P < 0.05). Additionally, compared to the CIH group, the escape latency of the IGF-1 + CIH group was shortened (^△^*P* < 0.05). The escape latency in the IGF-1 + CIH group was significantly higher than that in the NS + CIH group, and the difference was statistically significant (^#^*P* < 0.05). There was no significant difference between the CIH and the NS + CIH groups in escape latency

**Fig. 3:**
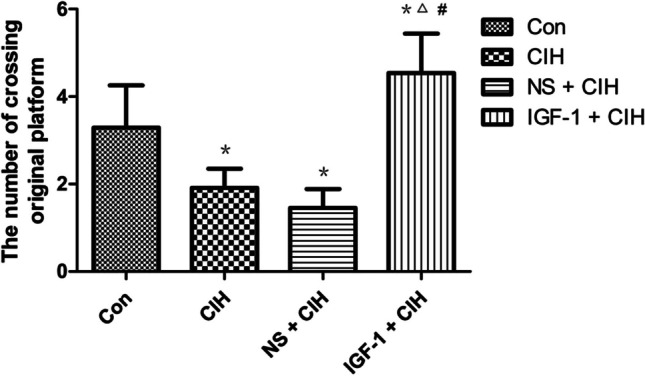
The number of rats crossing the platform in the space exploration test (*n* = 10, $\overline{x }$ ± s). Compared with the control group, the number of rats crossing the platform in the CIH group was significantly reduced, and the differences were statistically significant (^*^P < 0.05). Compared with the CIH group, the IGF-1 + CIH group significantly increased the number of rats crossing the platform, and the difference was statistically significant (^△^*P* < 0.05). The frequency of crossing the platform in the IGF-1 + CIH group was significantly higher than that in the NS + CIH group, and the difference was statistically significant (^#^*P* < 0.05). The CIH group and the NS + CIH group did not differ significantly

Through the observation of the space exploration experiment on the 7th d of the Morris water maze, we found that rats in the CIH group crossed the platform significantly less than those in the control group. There was no statistically significant difference between the IGF-1 + CIH group and the control group, despite the fact that it seemed like they crossed the platform more frequently (Fig. 3).

### IGF-1 can improve histopathological changes in the hippocampus of rats exposed to CIH

**Fig. 4:**
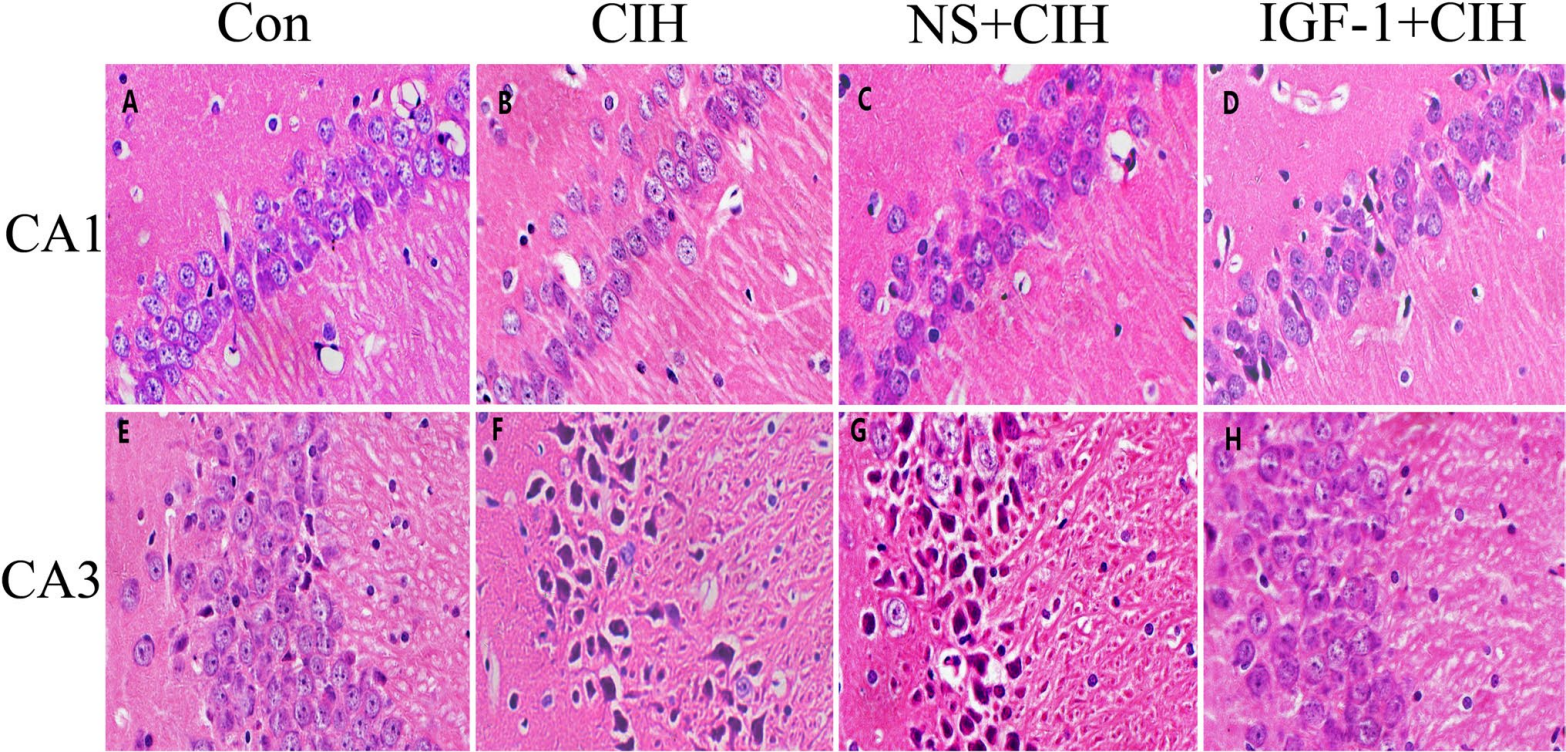
Effects of IGF-1 on pathological changes of the hippocampus in rats (*n* = 4, $\overline{x }$ ± s). **A**, **B**, **C**, and **D** The CA1 area of the rat hippocampi. **E**, **F**, **G**, and **H** The CA3 area of the rat hippocampi. **A** and **E** Control group. **B** and **F** CIH group. **C** and **G** NS + CIH group. **D** and **H** IGF-1 + CIH group. Each sample was repeated three times

In the hippocampus of rats in the control group, neuronal cells were neatly arranged and densely packed, with regular morphology, clear structure, lightly stained cytoplasm, centrally located nuclei, and prominent nucleoli (Fig. 4A and E). In the hippocampus of rats in the CIH group and the NS + CIH group, neuronal cells appeared disorganized and sparsely arranged, with irregular cell size and morphology, swollen and deformed cell bodies, wrinkled cell membranes, darkly stained cytoplasm, and nuclei that were hardened and fragmented (Fig. 4B-C, F-G). Rats in the IGF-1 + CIH group also had somewhat disordered hippocampal neurons with darker cytoplasm, though not as much as in the OSAS model and treatment control groups (Fig. 4).

### Subcutaneous injection of IGF-1 could restore decreased IGF-1 levels in the hippocampus of CIH rats

**Fig. 5:**
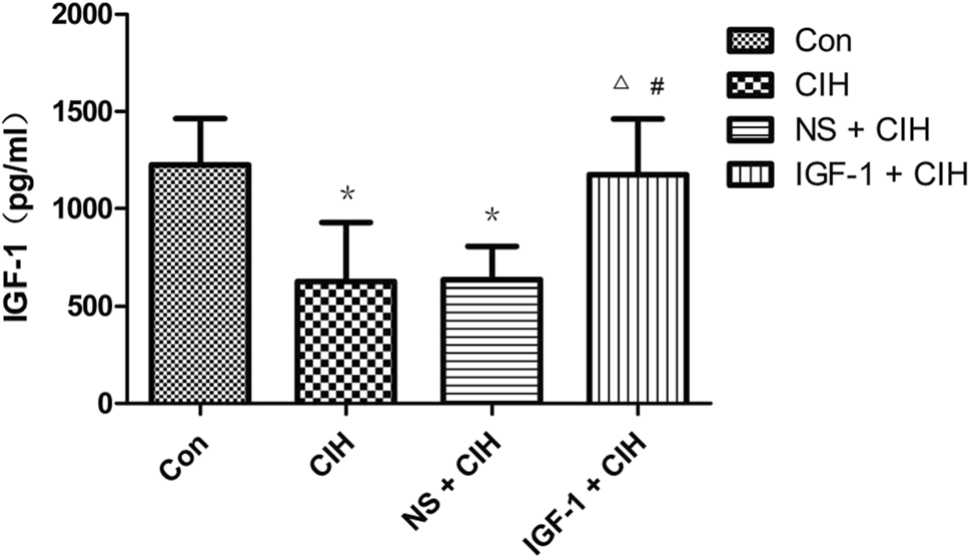
Expression of IGF-1 in the hippocampus of rats (*n* = 4, $\overline{x }$ ± s). The expression of IGF-1 in the hippocampus of rats exposed to CIH was less than the control group (^*^P < 0.05). Compared with the CIH group, the expression of IGF-1 in rats in the IGF-1 + CIH group was significantly increased, and the differences were statistically significant (^△^*P* < 0.05). The expression of IGF-1 in the hippocampus of rats in the IGF-1 + CIH group was significantly higher than that in the NS + CIH group, and the difference was statistically significant (^#^*P* < 0.05). Each sample was repeated three times

Compared with the control group, the level of IGF-1 protein in the hippocampus of the CIH group decreased significantly, and compared with the CIH model group and the control group, the level of IGF-1 protein in the hippocampus of the IGF-1 + CIH group increased significantly. There was no statistically significant difference in the amount of IGF-1 protein in the hippocampus between the IGF-1 + CIH group and the control group, nor was there a significant difference in the expression of IGF-1 protein between the CIH group and the IGF-1 + CIH group. (Fig. 5).

### p-IGF-1R and SYP expression were significantly improved in the hippocampus of rats exposed to CIH by subcutaneous injection of IGF-1

**Fig. 6:**
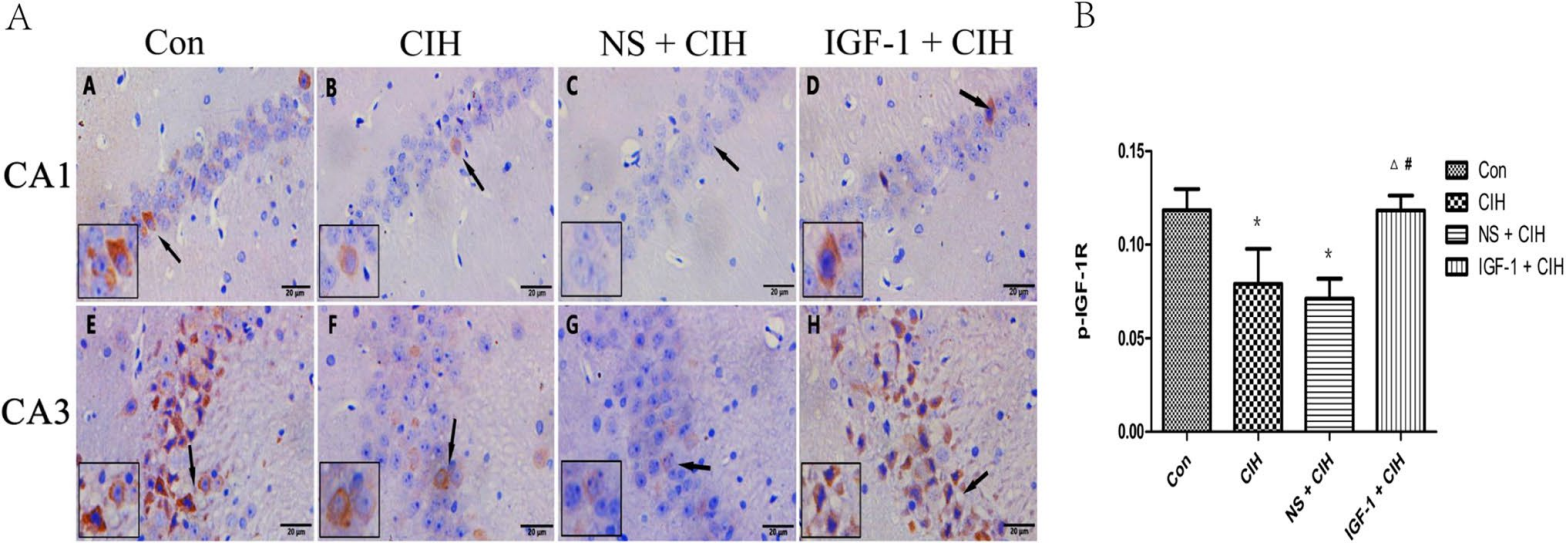
Localization and expression of P-IGF-1R in the hippocampus of rats. (*n* = 3, $\overline{x }$ ± s). **A** The CA1 and CA3 area of the rat hippocampus for each group. In panels A and E, the control group is represented. In panels **B** and **F**, the CIH group. In panels **C** and **G**, the NS + CIH group, and panels **D** and **H**, the IGF-1 + CIH group. **B** the quantitative analysis of p-IGF-1R expression. Each sample was repeated three times

**Fig. 7:**
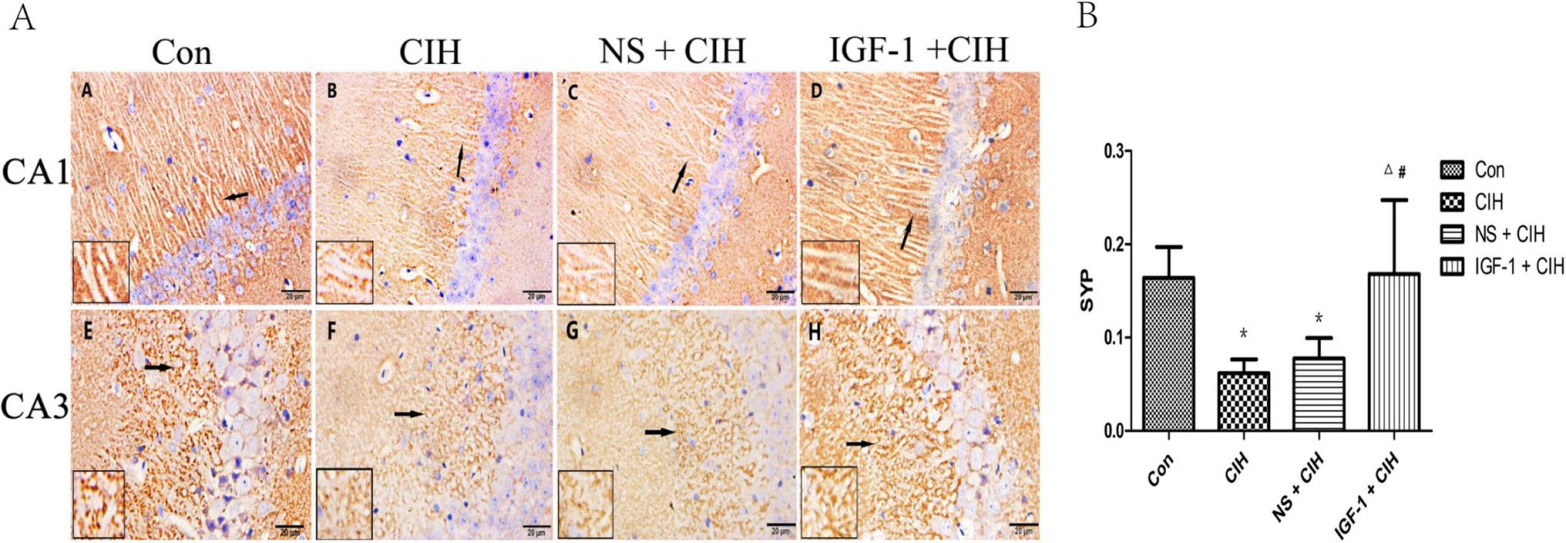
Localization and expression of SYP in the hippocampus of rats. (*n* = 3, $\overline{x }$ ± s). **A** The CA1 and CA3 area of the rat hippocampus for each group. In panels A and E, the control group is represented. In panels B and F, the CIH group. In panels C and G, the NS + CIH group, and in panels D and H, the IGF-1 + CIH group. **B** the quantitative analysis of SYP expression. Each sample was repeated three times

**Fig. 8:**
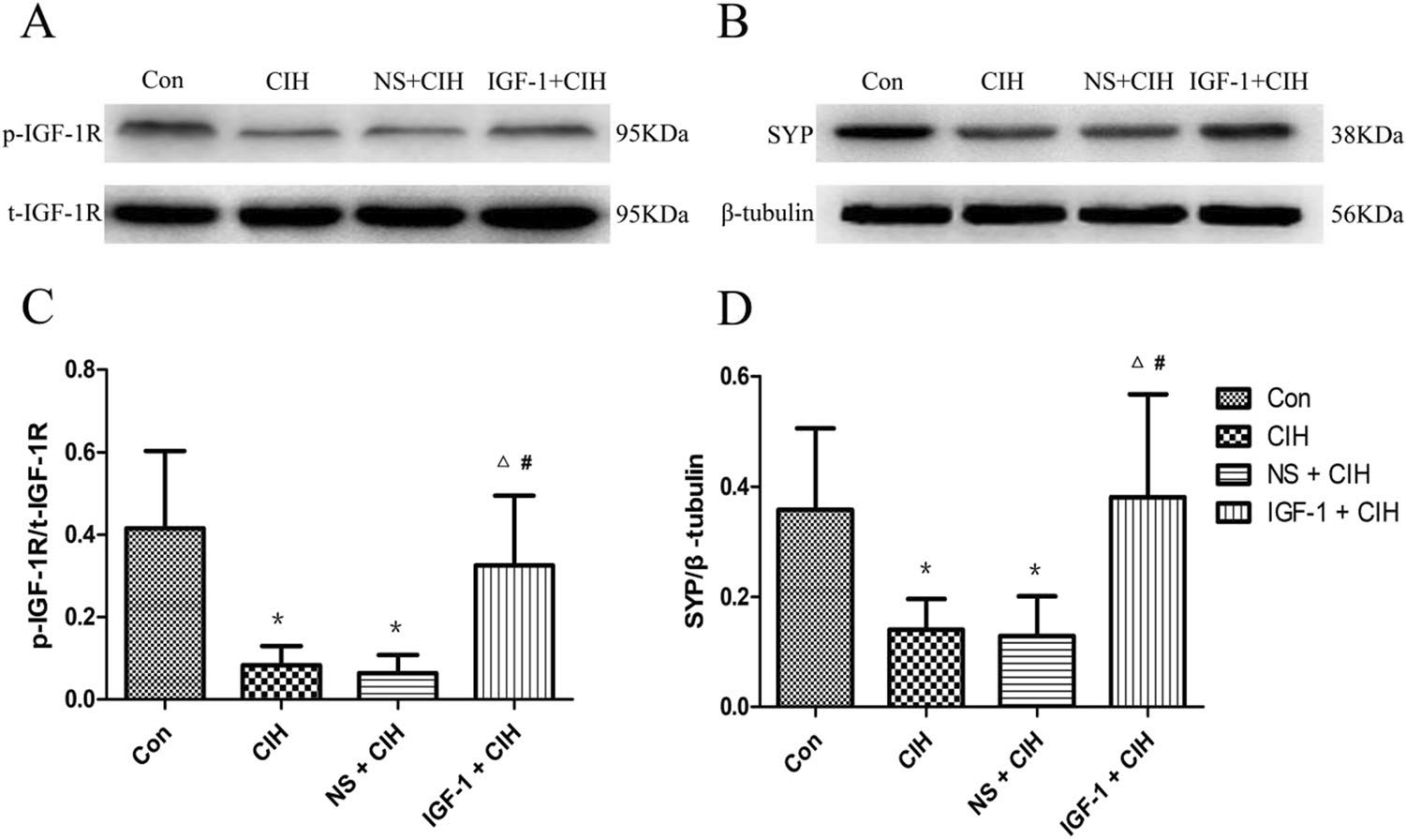
Expression of p-IGF-1R and SYP protein in the hippocampus of rats. (*n* = 6, $\overline{x }$±s). **A** WB representative band of p-IGF-1 and t-IGF-1R protein. **B** WB representative band of Synaptophysin and β-tubulin protein. **C** Relative expression of p-IGF-1R protein. **D** Relative expression of Synaptophysin. Compared with the control group, the expression levels of p-IGF-1R and SYP protein in the hippocampus of rats in the CIH group decreased, and the differences were statistically significant (^*^P < 0.05). Compared with the CIH group, the expression levels of p-IGF-1R and SYP protein in the hippocampus of rats in the IGF-1 treatment group were increased, and the differences were statistically significant (^△^*P* < 0.05). Each sample was repeated three times

IHC showed that the p-IGF-1R and SYP appeared as brownish-yellow hetero-staining granules in the hippocampus of rats. The p-IGF-1R mainly expressed in the neuronal envelope of the CA3 region, with a small amount also expressed in the CA1 region (Fig. 6A), while SYP primarily distributed on the neural protrusions in the CA1 and CA3 regions (Fig. 7A). The expression of p-IGF-1R and SYP in the hippocampus of rats in the CIH group was significantly lower than that in the control group (Figs. 6B and 7B). Nevertheless, CIH rats’ hippocampal p-IGF-1R and SYP expression rose dramatically following subcutaneous injection of IGF-1 (Fig. 8).

## Discussion

### CIH simulated OSAS animal model

OSAS is characterized as recurring total or partial collapse of the upper airway during sleep that causes hypoventilation or apnea. It is acknowledged as a highly common public health concern. It is characterized by CIH, sleep fragmentation (SF), hypercapnia, and frequently combined impairment of cognitive functions like memory, attention, executive function, and motor ability [11, 12]. The apnea–hypopnea index (AHI) and nocturnal oxygen saturation are currently the two clinical indicators most commonly used to evaluate the severity of OSAS [13]. There have been more lab studies on OSAS in recent years as society has given the condition more attention, but there still needs to be a set protocol for creating OSAS animal models at home or abroad. The surgical/mechanical model was the first to be used in laboratory research; it simulates the architecture of OSAS onset by mechanically blocking or changing the upper airway. However, because of the intrusive procedure, this model’s success rate is low [14].

The 2nd model, known as the SF model, is predicated on the idea that apnea or hypoventilation during sleep may cause frequent, fleeting awakenings, which would disrupt the regular architecture of sleep and reduce the total length of time that a person spends asleep [13], which might have a separate impact on the study’s findings [14]. Since the CIH model matches significant OSAS-related characteristics such as IH, arousal, sleepiness, and anxiety and is regarded to be more relevant to OSAS [13], it is commonly employed in research of OSAS mechanisms. It is believed that SF does not adequately capture the pathophysiological process of OSAS, whereas the CIH model does. In the meantime, numerous studies have demonstrated that CIH causes structural and functional changes in the rodent brain, including decreased synaptic plasticity, promoted middle cerebral artery occlusion, increased apoptosis of cortical and hippocampal neurons, promoted microglia activation, and elevated levels of β-amyloid (Aβ-42) in the brain [15, 16]. Additionally, CIH causes neuroinflammation [15], oxidative stress, reduced cerebral arterial blood flow [16], and other pathological processes [17, 18], all of which have been linked to OSAS cognitive impairment [11] and are therefore frequently used in research on OSAS cognitive impairment.

In this study, using the CIH model, the OSAS animal model was created, and the modeling methodology was enhanced by utilizing Julian [11] and Abdel-Wahab BA [19], among other sources. The rats were subjected to 180 s cycles of 20 hypoxic events/h for 8 h every d for 28 d. The end of the modeling period was used to measure the rats’ tail artery SpO_2_. When combined with the frequent awakenings, head-up breathing, snoring, and deepening of abdominal breathing, the rats’ SpO_2_ decreased by more than 4% during the hypoxic interval compared to the reoxygenation interval, which was consistent with the pathophysiological traits of OSAS.

### Effect of IGF-1 on the learning memory capacity of OSAS rats

Learning memory is a very complex higher neurophysiological activity of the brain involving several brain areas, multiple neurotransmitters, and intracellular signaling molecules [20, 21]. The hippocampus plays a critical role in this process. hippocampus regions CA1 [22] and CA3 [23] in particular. IGF-1, a newly discovered neurotrophic factor, can be produced locally in the brain or can cross the blood–brain barrier to enter the brain from the bloodstream [24]. It is important for maintaining normal brain function as well as for the process that prevents brain damage [25]. IGF-1 gene deficiency caused increased oxidative stress damage in mouse brain tissue, which was followed by brain edema and decreased learning and memory abilities. Clinical trials by Juanes [26] et al. showed that these symptoms improved following IGF-1 treatment. Survivors of traumatic brain injuries often develop persistent cognitive problems as a result of hippocampus atrophy. Exogenous IGF-1 supplementation has been demonstrated to improve cognitive dysfunction in TBI mice [10, 27]. Exogenous injection of IGF-1 decreased fluorouracil-induced neuronal death and increased cell proliferation, lowering cognitive dysfunction in rats [28]. Cognitive dysfunction was seen in rats given fluorouracil chemotherapy for 4 weeks. This experiment was carried out to determine if exogenous supplementation of IGF-1 could alleviate cognitive dysfunction in OSAS rats by subcutaneous injection of IGF-1 into the abdomen of OSAS rats based on the therapeutic potential of IGF-1 in cognitive disorders. A popular behavioral test used in cognitive science research to assess an animal’s ability to learn and remember spatial information is the Morris water maze. The Morris water maze was used in this experiment to test the spatial learning and memory skills of OSAS-affected rats and to look for signs of cognitive dysfunction in OSAS-affected rats. The impact of improving in the locomotor navigation test, the mean escape latency was significantly greater in the locomotor navigation test than in the blank control group, indicating a decline in spatial learning ability. In the spatial exploration test, the number of times a subject crossed the original target platform was also reduced, indicating a decline in memory ability. However, compared to rats in the blank control group and the treatment control group, the mean escape latency of rats treated with IGF-1 was shorter, and more rats crossed the initial target platform, suggesting that IGF-1 may improve rats’ ability to learn and remember spatial information. We, therefore, conclude that exogenous IGF-1 supplementation can enhance OSAS rats’ spatial learning and memory abilities.

### Effect of IGF-1 on the expression of p-IGF-1R and SYP in the hippocampus of OSAS rats

IGF-1R is a tetrameric structure (2α2β) made up of a transmembrane tyrosine kinase region (β subunit) and an extracellular cysteine-rich region (α subunit). When IGF-1 binds to the α receptor of IGF-1R, the transmembrane tyrosine kinase is phosphorylated, which in turn activates downstream signaling pathways [29]. The majority of researchers believe that circulating 1GF-1 can enter the brain through the blood–brain barrier (BBB) with IGF-1R, which is widely distributed in various brain regions and thus exerts a variety of neuroprotective effects [30]. Both cerebrovascular endothelial cells and choroid plexus epithelial cells in the BBB express large amounts of IGF-1R, and the uptake of IGF-1 in cerebrospinal fluid shows saturation kinetics, suggesting the presence of IGF transport carriers in the BBB [31, 32].

The hippocampal tissues of the rats were collected after each group had completed the behavioral test of the Morris water maze in this study, and the relative concentrations of IGF-1 in each group’s hippocampus were compared by ELISA. IGF-1 was subcutaneously injected into the abdomen of OSAS-affected rats. The experiment’s findings showed that OSAS may cause rats’ hippocampi to express less IGF-1. The lowered IGF-1 level in the hippocampus of OSAS-affected rats may be reversed by subcutaneous abdominal injection of IGF-1, which may have occurred because IGF-1 crossed the blood–brain barrier to enter the hippocampus from the subcutaneous abdominal injection.

One of the crucial brain regions is the hippocampus, whose synaptic organization and plasticity are strongly associated with spatial learning and memory; CIH is a pathophysiological process that is distinctive to OSAS and is crucial to the onset of OSAS [33]. The density and distribution of SYP, which is highly concentrated in dendritic spines and is intimately associated with cognitive function, can serve as an indirect indicator of the quantity and distribution of synapses in vivo [4]. As brain development progresses, SYP finally goes to the synapse to produce a punctate distribution pattern, indicating that SYP is directly associated with synapse formation and maturation [34]. SYP is initially mostly distributed in the nucleus of neurons. After hippocampal neurons die, hippocampal pyramidal cells can extend spine protrusions to reach adjacent spine terminals and form new synapses. This process is stable and requires the synaptic protein SYP, which improves the effectiveness of hippocampal synaptic reconstruction [17]. SYP simultaneously influences synaptic structure and neurotransmitter release, which modulates synaptic plasticity [17, 35]. Hippocampal SYP expression is frequently observed to be diminished in animal models of cognitive deficits [18, 36], whereas an increase in hippocampal SYP expression frequently accompanies cognitive recovery [35, 37]. Hippocampal synapses undergo considerable structural and functional changes following OSAS [38]. However, unknown processes underlie the changed expression of SYP protein in cognitive impairment associated with OSAS.

The results of this investigation showed that in rats affected by OSAS, hippocampus p-IGF-1R, and SYP protein levels were considerably reduced by OSAS; however, these levels were recovered by IGF-1 therapy. This finding was consistent with the altered spatial learning and memory function of the rats, indicating that the decreased spatial learning and memory function of OSAS-affected rats was closely related to the down-regulation of hippocampal IGF-1R activation le.

According to the results of the study mentioned above, IGF-1 may protect OSAS rats’ spatial learning memories by activating IGF-1R and upregulating SYP expression in the hippocampal region. We were able to establish the significance of IGF-1 in synaptic modulation and its potential as a prophylactic drug for cognitive dysfunction associated with OSAS, even if this experiment was unable to pinpoint the signaling pathways involved in this process.

## Data Availability

The datasets generated during and/or analyzed during the current study are available from the corresponding author on reasonable request.
